# Caspase 3-specific cleavage of ubiquitin-specific peptidase 48 enhances drug-induced apoptosis in AML

**DOI:** 10.1080/15384047.2025.2459426

**Published:** 2025-01-29

**Authors:** Zhanglin Zhang, Xiang Lin, Yaling Yang, Xuemei Wang, Yi Wang, Xianbao Huang, Miao Hong, Wei Gao, Hua He, M. James You, Yi Yang, Guangyao Kong

**Affiliations:** a National & Local Joint Engineering Research Center of Biodiagnosis and Biotherapy, Department of Hematology, Precision Medical Institute, The Second Affiliated Hospital of Xi’an Jiaotong University, Xi’an, Shaanxi, China; b Departments of Blood Transfusion, Institute of Transfusion, Jiangxi Key Laboratory of Transfusion, The First Affiliated Hospital, Jiangxi Medical College, Nanchang University, Nanchang, China; c Department of Hematopathology, The University of Texas, MD Anderson Cancer Center, Houston, TX, USA; d Department of Hematology, The First Affiliated Hospital, Jiangxi Medical College, Nanchang University, Nanchang, China

**Keywords:** AML, apoptosis, deubiquitination, USP48, caspase 3

## Abstract

Dysfunction or dysregulation of deubiquitination is closely related to the initiation and development of multiple cancers. Targeted regulation of deubiquitination has been recognized as an important strategy in tumor therapy. However, the mechanism by which drugs regulate deubiquitinase is not clear. Here, we identified ubiquitin-specific peptidase 48 (USP48), a member of the ubiquitin-specific protease family highly expressed in various tumors, as a specific substrate for the activated caspase-3. During drug induced apoptosis of AML cells, activated caspase-3 cleaves USP48 through recognizing the conservative motif DEQD located at 611–614 sites of human USP48. Subsequent analysis showed that the cleavage USP48 N-terminal fragment which contains catalytic active domain is easily degraded by ubiquitination. Meanwhile knockdown experiment showed that inhibiting the expression of USP48 could also promotes apoptosis and enhance the efficacy of chemotherapy drugs. Altogether, these results suggest that targeting USP48 may represent a novel therapeutic strategy in AML.

## 1. Introduction

Acute myeloid leukemia (AML) is the most common form of acute leukemia in adults, with an increasing incidence as people age.^1^ This genetically heterogeneous malignancy is characterized by the clonal expansion of abnormal myeloid progenitor cells.^2^ Despite advances in standard chemotherapy, such as the “3 + 7 regimen,” and novel treatment strategies, most patients still face poor prognoses.^2–4^ Therefore, new therapeutic strategies are urgently needed.

Ubiquitination, the process of adding ubiquitin to target proteins, plays a critical role in protein stability, localization, interactions, and activity, influencing cell fate decisions such as survival, death, differentiation, and immune regulation.^5^ Deubiquitinases (DUBs) hydrolytically remove ubiquitin from protein adducts.^6^ Dysfunction or dysregulation of ubiquitination and deubiquitination is closely related to the initiation and development of multiple cancers including AML.^7–9^ In humans, ubiquitin-specific proteases (USPs) are the most numerous classes of DUBs, with approximately 60 members.^10^ USP48, a member of this family, was initially reported to protect NHE3 from degradation, thereby regulating blood pressure and sodium balance.^11^ Subsequent research revealed that USP48 promotes tumor progression, metastasis, and chemoresistance by inhibiting the degradation of cancer-related proteins such as MDM2, RelA, Gli1, Aurora B, and HMGA2.^12–16^ Suppression of USP48 inhibits proliferation and promotes tumor cell death, making it a potential target for cancer therapy.

PR-619, a broad DUB inhibitor that includes USP48, is considered a promising anti-cancer drug.^17^ Studies have shown that PR-619 induces apoptosis and autophagy through ubi-protein aggregation-activated ER stress in esophageal squamous cell carcinoma.^18^ It can also enhance the cytotoxic and apoptotic effects of cisplatin by suppressing c-Myc in cisplatin-resistant urothelial carcinoma cells.^19^ Recently, PR-619 was found to induce ferroptosis and trigger antitumor immunity in colon cancer cells.^20^ However, little is known about effects and mechanism of PR-619 in AML.

In this study, we demonstrated that PR-619 reduces USP48 protein levels by promoting its cleavage in various leukemia cells. Mechanistically, we discovered that caspase-3 recognizes a conserved DEQD motif located at amino acids 611–614 of human USP48 and cleaves USP48 during drug-induced apoptosis. Further experiments showed that the N-terminal fragment of cleaved USP48 is easily degraded by ubiquitination. Additionally, inhibiting USP48 expression significantly reduces AML cell colony formation, promotes apoptosis, and enhances the efficacy of chemotherapy drugs. These findings suggest that targeting USP48 may offer a novel therapeutic strategy for AML.

## 2. Materials and methods

### 2.1. Reagents and antibodies

DUB inhibitor PR-619 (HY-13814), Homoharringtonine (HHT, HY-14944), Cytarabine (Ara-C, HY-13605), all-trans-Retinoic acid (ATRA, HY-14649), Z-DEVD-FMK (HY-12466) and Cell Counting Kit-8 (HY-K0301) were obtained from MedChem Express. Cycloheximide (CHX, 66-81-9) and Z-Leu-Leu-Leu-al (MG132, C2211) were from Sigma-Aldrich company. The recombinant caspase-3 protein (ab52101) was purchased from Abcam Inc. The antibodies against PARP (#9542, 1:1000), and Cleaved Caspase-3 (#9661, 1:1000) were purchased from Cell signaling technology, Inc. The β-actin (81115–1-RR, 1:5000), flag (66008–4-Ig, 1:5000) and USP48 antibody (12076–1-AP, 1:1000) was purchased from Proteintech Group, Inc. Annexin-V-FITC/PtdIns kit was purchased from Bestbio Biotechnology (Bestbio, China). The lentiviral expression vector pLVX-IRES-zsGreen, pLVX-shRNA1-Puro vector and the lentiviral packaging plasmids were provided by Clontech Laboratories, Inc.

### 2.2. Cell culture and treatment

The AML cell lines U937, NB4, OCI-AML2, KG-1, HEL cells were derived from James You Laboratory in the MD Anderson Cancer Center at the University of Texas in the United States. All the cells maintained in RPMI-1640 containing 10% fetal bovine serum under 37°C with 5% CO_2_. HEK 293T cells cultured in DMEM containing 10% fetal bovine serum under 37°C with 5% CO_2._

### 2.3. Plasmid construction, transfection and lentiviral transduction

The USP48(FL, N610, C420) were cloned in-frame to the flag tag at the C-terminal to generate the pLVX-USP48 (FL)-IRES-zsGreen，pLVX-USP48(N610)-IRES-zsGreen, pLVX-USP48 (C420)-IRES-zsGreen. Two efficient shRNAs targeting human USP48 (sh2: GCGTAAGCAAAGTGTGGATAA, sh3: GAATCCAGATGTGCGCAATAT) were synthetized and cloned into the lentiviral vector pLVX-shRNA1-Puro. The plasmids were transfected into 293T cells together with the packing plasmids pSPAX2 and pMD2G based on the manufacturer’s protocol. A total of 48 h post-transfection, virus was harvested and used to infect AML cells.

### 2.4. Cell proliferation analysis (CCK8 assay)

Cells (1 × 10^3^) per well were seeded into 96-well plates in 100 μl volume and cultivated in RPMI1640 medium with 10% fetal bovine serum at 37°C. Cells were grown for 24, 48 and 72 hours, respectively. 10 μl of CCK8 reagent was added and incubated in 37°C for 3 hours. The absorbance value of 450 nM was measured, and analyzed the proliferation ability of cells.

### 2.5. Immunoblotting analysis

Immunoblotting analysis was performed as described previously.^21^ Briefly, Proteins from cell lysates (20 µg) were separated with 10% SDS-PAGE and electrotransferred to nitrocellulose (NC) membranes. The NC membranes were sealed with 5% nonfat dry milk and blotted with primary antibody overnight at 4°C. Then membranes were incubated with peroxidase-conjugated secondary antibody and washed with TBST three times, proteins were imaged by enhanced chemi-luminescence detection reagent and detected with Bio-Rad ChemiDoc XRS+ chemiluminescence imaging system (Bio-rad laboratories Inc.)

### 2.6. Apoptosis analysis by flow cytometry

Apoptotic cells were detected using an annexin V- fluorescein isothiocyanate (FITC) Kit (MBL Life Science) according to the manufacturer’s instructions. Briefly, cells were collected, resuspended in the binding buffer supplemented in the kit, incubated with annexin V-FITC in the dark for 15 min, and then added PI for 5 min. Stained cells were analyzed using a flow cytometer (BD FACSCanto^TM^ II, Becton Dickinson, USA).

### 2.7. Cell cycle analysis

Cells (1 × 10^6^) were harvested and then stained using the BD Accuri C6 PI staining Reagents kit (Becton Dickinson, USA), according to the manufacturer’s protocol. Stained cells were analyzed using a flow cytometer (BD FACSCanto^TM^ II, Becton Dickinson, USA) and cell cycle analysis was performed using MODFIT LT 4.1 software (Verity Software House).

### 2.8. Colony formation assays

U937 cells were infected with lentivirus expressing shRNA against USP48 for 48 h (scrambled shRNA as control). After selecting with puromycin for 48 h, transduced cells were counted and plated in 1.2% methylcellulose RPIM 1640 medium supplemented with 100 IU/mL penicillin and 100 μg/mL streptomycin, 10% FBS with 2,000 cells per well in 12-well culture plates. The cells were stained by adding 5 mg/ml of MTT reagent after 14 days of incubation. Colonies were detected and scored with the Image J quantification software.

### 2.9. In vitro USP48 cleavage assay

The mutant USP48 with D614A was cloned into pLVX-IRES-zsGreen vector. 293T cells were transfected with wild type or D614A USP48 for 24 h, and the cells were washed once in PBS and resuspended in lysis buffer (50 mm Tris-HCl pH 7.4, 100 mm NaCl, 1 mm EDTA, 10% glycerol and 0.1% NP-40, 0.1 mm PMSF). Caspase-3 treatment was performed as previously described with some modifications.^22^ Briefly, 20 μg of protein in lysis buffer was diluted 1:10 (V/V) in caspase buffer (50 mm HEPES pH 7.4, 100 mm NaCl, 1 mm EDTA, 10% glycerol and 0.1% NP-40). Two units of caspase-3 (ab52101, Abcam Inc., Toronto, ON, Canada) were added, and the samples were rotated for 2 h at 37°C. protein loading buffer (5×) was added, and the samples were boiled for 10 min. USP48 cleavage was analyzed with western blot.

### 2.10. Statistical analysis

Statistical analyses were performed using GraphPad Prism 6 (GraphPad Software, Inc.). Student’s t-test was used to compare the differences between treatment and control groups. ANOVA was used to analyze the differences in proliferation and cell cycle of the cells. Following ANOVA, Bonferroni’s post hoc test was used to determine significant differences. All data are presented as mean ± standard deviation. *p* < .05 was considered to indicate a statistically significant difference. *, **, ***, **** represent *p* < .05, 0.01, 0.001, 0.0001, respectively.

## 3. Results

### 3.1. USP48 cleavage in drug induced apoptosis of AML

**Figure 1.**
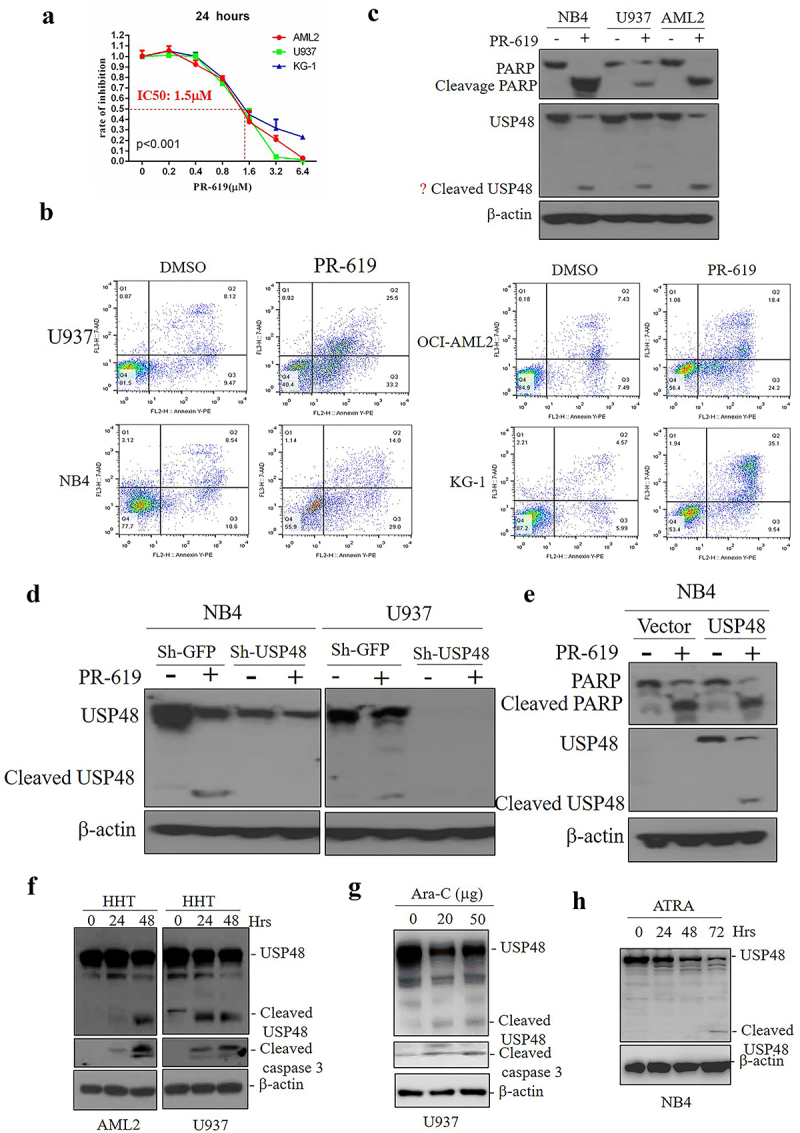
USP48 cleavage in DUB inhibitor PR-619 induced apoptosis of AML cells. (a) The IC50 of PR-619 in the AML cell line after 24 hours with treatment indicated concentrations, *n* = 5. (b) Apoptosis analysis of U937, OCI-AML2, NB4, and KG-1 cells following treatment with 2 μM PR-619 for 24 h. *n* = 3. (c) Western blot of the cleavage PARP and USP48 in NB4, U937, AML2 cell lines with or without the treatment with 2 μM PR-619 for 24 h. (d) Western blot of the cleavage USP48 in NB4 and U937 cell lines with USP48 knockdown, and treated with 2 μM PR-619 for 24 h. (e) Western blot of the cleavage PARP and USP48 in NB4 cell line overexpressing USP48 and treated with 2 μM PR-619 for 24 h. (f) Western blot of cleavage PARP and USP48 in AML2 and U937 cell lines with treatment at indicated time points followed by 30 nM HTT treatment. (g) Western blot of cleavage PARP and USP48 in U937 cell line treated with indicated concentrations (0, 20, 50 μg/mL) Ara-C for 24 h. (h) Western blot of cleavage USP48 in NB4 cell line with treatment at indicated time points followed by 1 μM ATRA treatment.

We first assessed the efficacy of PR-619 on AML cell lines. PR-619 inhibited cell proliferation in a dose-dependent manner (Figure 1a) and induced apoptosis in U937, OCI-AML2, NB4, and KG-1 cells (Figure 1b). Western blot analysis revealed that PR-619 reduced USP48 protein levels and promoted PARP cleavage (Figure 1c). Notably, an additional band around 55 kD appeared after PR-619 treatment, detected by the USP48 antibody, which disappeared following USP48 knockdown (Figure 1d). Overexpression of flag-tagged USP48 in NB4 cells also showed that PR-619 induced cleavage of exogenous USP48-flag protein (Figure 1e). Additionally, USP48 cleavage was observed in AML cells treated with chemotherapy drugs HHT (Figure 1f) and Ara-C (Figure 1g), and during ATRA-induced differentiation in NB4 cells (Figure 1h), indicating that both PR-619 and chemotherapy drugs induce USP48 cleavage in AML.

### 3.2. USP48 is specifically cleaved by activated caspase-3

**Figure 2.**
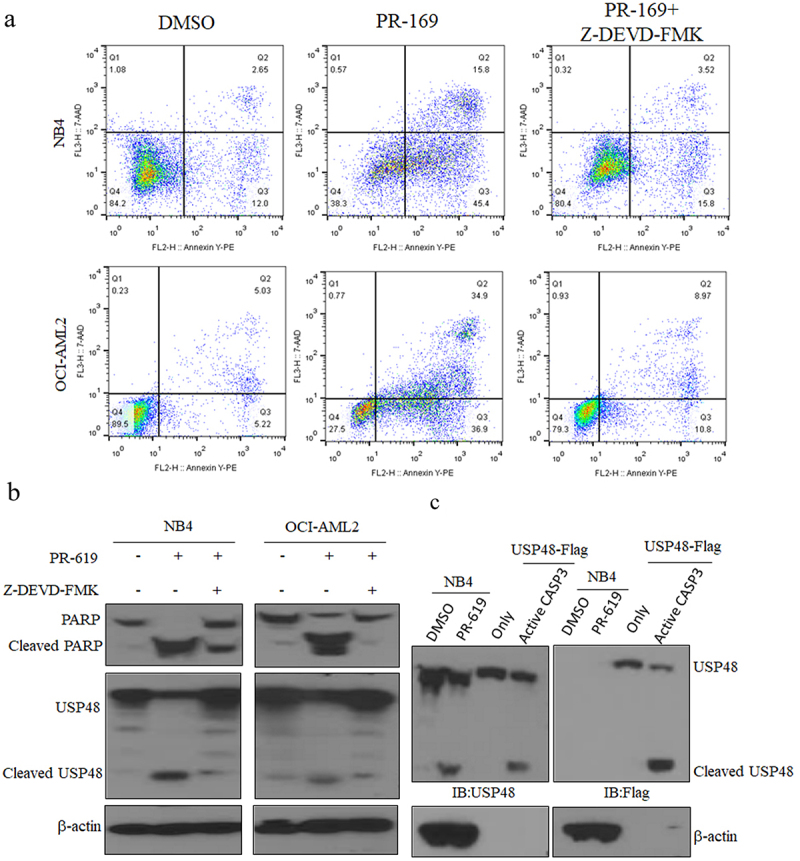
USP48 is specifically cleaved by activated caspase-3. (a-b) apoptosis analysis (a) or western blot (b) of cleavage PARP and USP48 in NB4 and OCI-AML2 cell lines treated with PR-169 (2 μM) alone or in combination with caspase-3 specific inhibitor Z-DEVD-FMK (50 μM) for 2 h. (c) Western blot of cleavage USP48 from by activated caspase-3 cleaved USP48 with in vitro cleavage assay.

We hypothesized that PR-619 and chemotherapy drugs induce USP48 cleavage through activated caspase-3. To test this, we used the caspase-3 specific inhibitor Z-DEVD-FMK, which was added 2 hours before PR-619 treatment. Flow cytometry analysis shown that Z-DEVD-FMK inhibited apoptosis of NB4 and OCI-AML2 cells induced by PR-619 (Figure 2a). Western blot analysis confirmed that the caspase-3 inhibitor reduced PARP and USP48 cleavage after PR-619 treatment (Figure 2b). In vitro experiments demonstrated that activated caspase-3 cleaved USP48-flag, producing a ~ 55 kD fragment detected by both USP48 and flag antibodies (Figure 2c). These data suggest that USP48 is cleaved by activated caspase-3 during apoptosis.

### 3.3. USP48 cleavage occurs at an evolutionarily conserved DEQD motif

**Figure 3.**
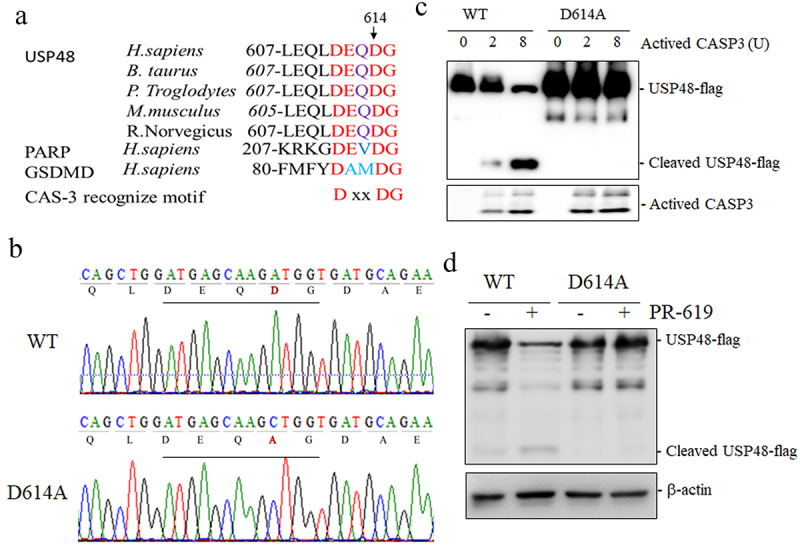
USP48 cleavage occurs at an evolutionarily conserved DEQD motif. (a) An evolutionarily conserved DEQD motif locates at 611–614 sites of mammals USP48. (b) The sanger sequence results of the mutation of the aspartic acid at position 614 to alanine in USP48 conserved DEQD motif. (c) Western blot of cleavage USP48 from by activated caspase-3 cleaved USP48 with in vitro cleavage assay, the cell lysis of the HEK293T transfected USP48 WT or D614A were incubated with activated 2 units caspase-3 at 37°C or 2 hours in vitro. (d) Western blot of cleavage USP48 in HEK293T overexpression USP48 WT or D614A treated with 10 μM PR-619 for 12 h.

To identify the cleavage site on USP48, we compared caspase-3 cleavage motifs on PARP and GSDMD and found a conserved DEQD motif at amino acids 611–614 in USP48 (Figure 3a). We hypothesized that caspase-3 cleaves USP48 after this residue. To test this, we mutated the aspartic acid at position 614 to alanine (D614A) and performed in vitro cleavage analysis with activated caspase-3 (Figure 3b). The mutation prevented the generation of the cleavage fragment in the mutant USP48 group, unlike in the wild type (Figure 3c). PR-619 also failed to cleave the mutant in NB4 cells (Figure 3d). Thus, the DEQD motif is the caspase-3 cleavage site in USP48.

### 3.4. N-terminal of cleaved USP48 is unstable due to proteasome-mediated degradation

**Figure 4.**
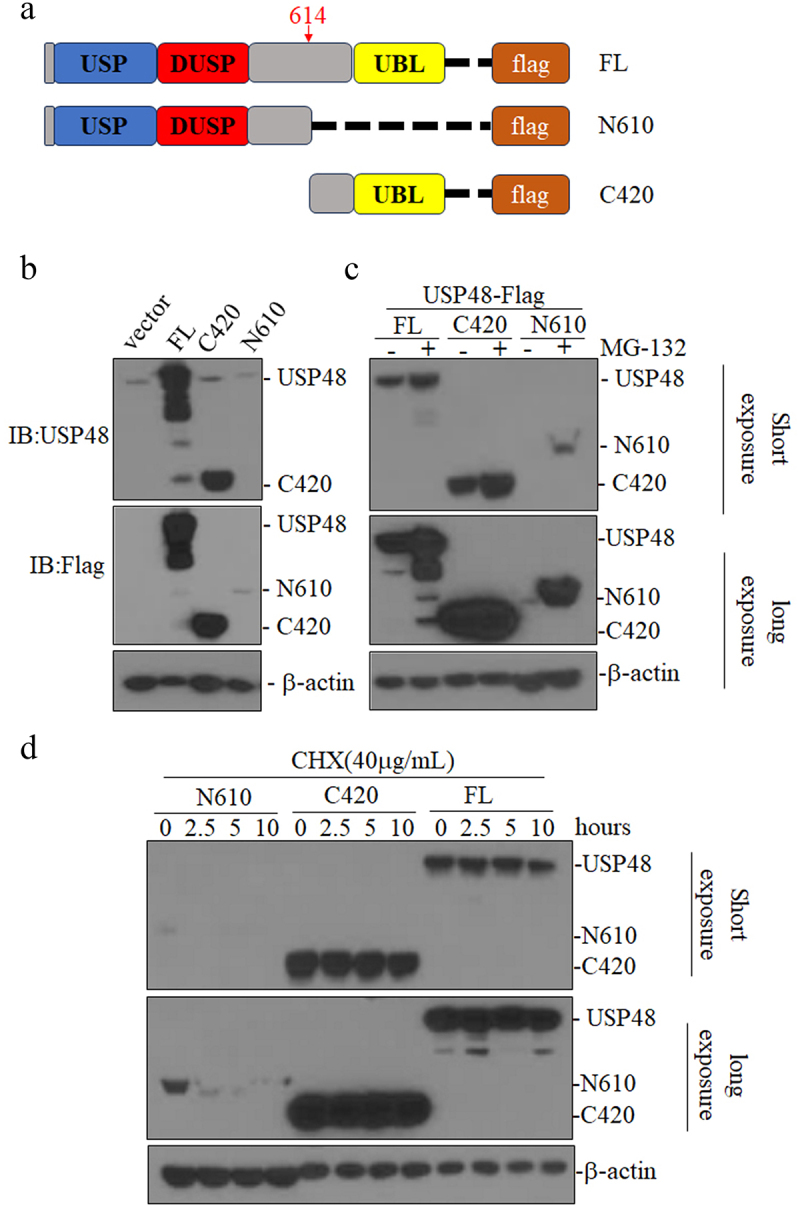
N-terminal of cleaved USP48 is unstable by proteasome-mediated-degradation. (a) The pattern diagram of the USP48 (FL, N610 and C420) fused with flag tag. (b) West blot of the USP48-FL, USP48-N610 and USP48-C420 in 293T with anti-USP48 and anti-flag antibody. (c) Western blot of the USP48-C420 in HEK293T (d) Western blot of USP48 from USP48 FL, N610, or C420-HEK293T treated with or without 10 μM MG132 for 12 hours. (e) Western blot of USP48 from USP48 FL, N610, or C420-HEK293T treated with 40 μg/mL CHX at indicated time points.

To explore the function of USP48 cleavage fragments in AML, we constructed lentiviral expression plasmids for N-terminal (N610) containing UCH and DUSP domains, and C-terminal (C420) containing the UBL domain of USP48, fused with a flag tag (Figure 4a). We then overexpressed N610 and C420 in HEK 293T and detected with USP48 and flag antibody. We found USP48 antibody could detected C420 only and flag antibody could detect N610 and C420, but N610 protein is expressed very low (Figure 4b). The molecular weight of C420 matched the endogenous cleavage fragment of USP48 (Figure 4c). Treatment with MG132 significantly increased N610 protein levels (Figure 4d), After inhibiting protein synthesis with CHX, USP48-FL and C420 remained stable, while N610 levels decreased by more than 90% after 2.5 hours (Figure 4e), indicating that the cleaved N-terminal fragment of USP48 is rapidly degraded by ubiquitination.

### 3.5. Knockdown of USP48 reduces colony formation, induces apoptosis and G1 phase arrest in AML cell lines

**Figure 5.**
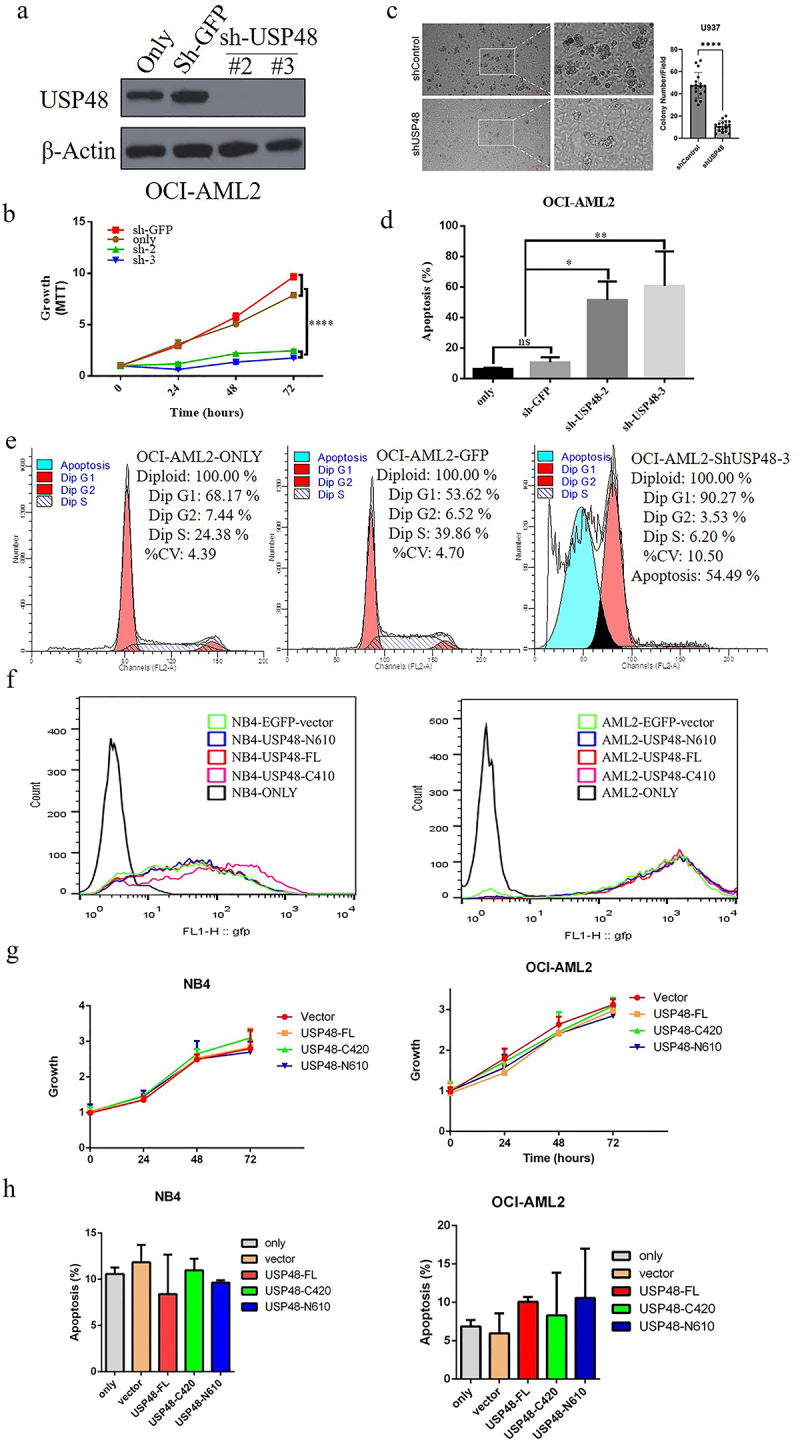
Knockdown of USP48 induces apoptosis and G1 phase arrest in OCI-AML2 cells. (a) Western blot of USP48 in OCI-AML2 transfected shGFP, shUSP48#2, shUSP48#3. (b) CCK-8 assay analyzed growth curves of OCI-AML2 cell lines transfected with shGFP and shUSP48. (c-e) colony formation (c), apoptosis (d) and cell cycle (e) analysis of OCI-AML2 cells liens transfected shGFP, shUSP48#2, shUSP48#3. (f) Flow cytometry analysis of infection efficiency of control, USP48 FL, USP48-C420, USP48-N610 in NB4 and OCI-AML2 cell lines. (g) CCK-8 assay analyzed growth curves of NB4 and OCI-AML2 cell lines transfected with control, USP48 FL, USP48-C420, USP48-N610. (h) Apoptosis analysis of NB4 and OCI-AML2 cell lines transfected with control, USP48 FL, USP48-C420, USP48-N610.

To examine the proliferation effect of USP48 in AML, USP48 was ectopically repressed with shRNAs in OCI-AML2 (Figure 5a). Compared with cell only and vector control, USP48 knockdown in OCI-AML2 significantly reduced cell proliferation (Figure 5b) and colony formation (Figure 5c), promoted apoptosis (Figure 5d) and induced G1 phase arrest (Figure 5e). Similarly, the inhibition effects also found in the AML cells lines NB4 and HEL (data not show). We also overexpressed N610 and C420 in NB4 and OCI-AML2, and Analyzed the impact of USP48 cleavage fragments on AML. Flow cytometry analysis showed that the expression efficiencies of USP48-FL, N610 and C420 were all above 90% in NB4 cells and AML cells (Figure 5f). However, overexpression did not affect AML cell proliferation and apoptosis (Figure 5g,h). All the data indicated that inhibition of USP48 expression could decrease growth of AML cells by inducing apoptosis and G1 phase arrest.

### 3.6. Inhibition of USP48 enhances the sensitivity of AML cells to chemotherapy drug

**Figure 6.**
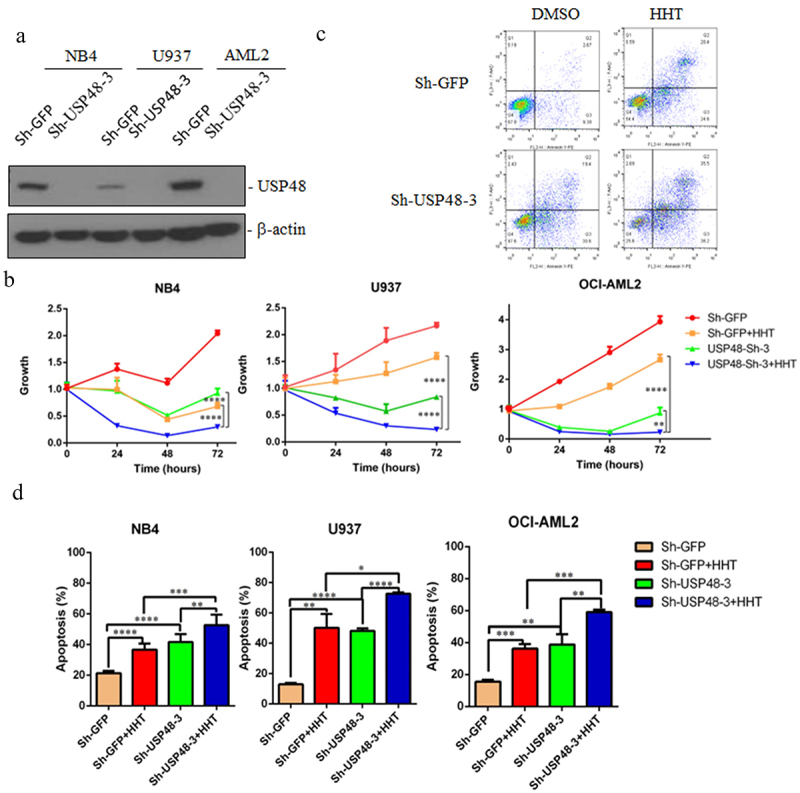
Inhibition of USP48 enhances the sensitivity of AML cells to HHT. (a) Western blot of the USP48 in NB4, U937, and OCI-AML2 cell lines transfected shGFP, shUSP48#3. (b) CCK-8 assay analyzed growth curves of NB4, U937, and OCI-AML2 cell lines with USP48 knockdown, and treated with the treatment with HTT (30 nM for 24 hours). (c) Apoptosis analysis of NB4 cells lines transfected with shGFP, shUSP48#3 with the treatment with HTT (30 nM for 24 hours). (d) Statistical analysis of apoptosis of NB4, U937 and OCI-AML2 with USP48 knockdown, and treated with the treatment with HTT (30 nM for 24 hours).

We continued to analyze the effect of inhibiting USP48 on AML chemotherapy. We inhibited USP48 expression by shRNA in NB4, U937, and OCI-AML2 cells (Figure 6a), and further analyze its impact on the treatment of chemotherapy drug HHT. MTT experiments showed that both HHT and USP48 shRNA could inhibit AML cell proliferation, and inhibiting USP48 could enhance the effect of HHT (Figure 6b). Flow cytometry analysis also showed that both HHT and USP48 shRNA induced apoptosis of AML cells, HHT combined with USP48 shRNA enhances cell apoptosis (Figure 6c,d). All the data indicate that inhibition of USP48 enhances the sensitivity of AML cells to chemotherapy.

## 4. Discussion

In this study, we demonstrated that USP48 is specifically cleaved by caspase-3 during drug-induced apoptosis and differentiation in AML cells, thereby enhancing the apoptotic response to chemotherapy. These findings offer significant insights into the molecular mechanisms of AML treatment and highlight the potential of targeting USP48 as a therapeutic strategy.

As a member of the deubiquitinating enzyme family, USP48 plays a critical role in protecting target proteins from degradation by removing ubiquitin modifications. Previous studies have shown that USP48‘s deubiquitinating activity regulates NFκB signaling by stabilizing RelA in the nucleus, which is essential for cancer cell survival.^13,23^ Additionally, USP48 has been shown to promote tumorigenesis by stabilizing oncoproteins such as Gli1, TRAF2, and Mdm2.^12,16,24^ Beyond its oncogenic functions, USP48 is also involved in cell cycle progression,^14^ and its high expression levels in various tumors are closely associated with poor prognosis.^15,25^ Our study demonstrated that USP48 is a highly specific substrate of activated caspase-3. Caspase-3 recognizes and cleaves USP48 at the evolutionarily conserved DEQD motif, resulting in the instability of the N-terminal fragment. This cleavage sensitizes AML cells to apoptotic differentiation and death, indicating a critical role for USP48 in modulating the apoptotic response in AML.

Caspase-3 is a widely expressed member of a conserved family of proteins, generally recognized for their activated proteolytic roles in the execution of apoptosis in cells responding to specific extrinsic or intrinsic inducers of this mode of cell death.^26^ Apoptotic stimuli trigger the activation of the initiator caspases (caspase-2, −8, −9, and −10), which then cleave and thereby activate the effector members (caspase-3, −6, and −7).^27^ The latter, in turn, target and cleave proteins that contain the Asp-Glu-Val-Asp (DEVD) sequence motif such as PARP, ICAD, ROCK1. Besides DEVD, caspase-3 also recognizes VESD and VEGD motif.^28,29^ In our study, we found that a highly conserved DEQD sequence located at the 611–614 amino acid residues in the USP48 protein of mammals. Mutation of the aspartic acid residue at position 614 to alanine (D614A) abolished the cleavage of USP48 by caspase-3, confirming the specificity of this cleavage site.

The USP48 protein is highly conserved in mammals which contains a highly conserved ubiquitin-specific protease (USP) domain and deubiquitin-specific protease (DUSP) domain at the N-terminus and a UBL domain at the C-terminus.^30,31^ The USP domain is the catalytic domain that remove ubiquitin molecules from polyubiquinated peptides by cleavage of isopeptide bonds.^31^ The DUSP has a potential role in protein/protein interaction or substrate recognition.^32^ The UBL domain catalyzes the formation of ubiquitin-protein conjugates, and target ubiquitinated proteins for degradation.^33^ Interestingly, our data reveal that the N-terminal fragment of cleaved USP48 containing UCH and DUSP domains is highly unstable and undergoes proteasome-mediated degradation. This instability likely inhibits the deubiquitinase activity of USP48. Furthermore, inhibition of USP48 expression through shRNA knockdown in AML cell lines significantly reduced cell proliferation, promoted apoptosis, and induced G1 phase arrest. These effects were also observed with the use of chemotherapy drugs such as HHT, Ara-C, and ATRA, which induced the cleavage of USP48, thereby enhancing drug-induced apoptosis. The results indicate that caspase-3 cleavage disrupts the deubiquitination function of USP48 and inhibit growth of AML cells.

Another key finding of this study is the enhancement of chemotherapy efficacy through the inhibition of USP48. By combining USP48 knockdown with chemotherapy drug treatment, we observed a significant increase in AML cell apoptosis compared to either treatment alone. This suggests that targeting USP48 can potentiate the effects of existing chemotherapeutic agents, providing a synergistic approach to AML therapy. However, our study is entirely based on an in vitro experimental model, which may not fully reflect the complexity of the in vivo environment, and its clinical relevance still needs further verification. In the future, we will use animal models and clinical trials to explore the role of USP48 in the occurrence and development of AML, and further evaluate the potential value of USP48 specific inhibitors in AML treatment with the PDX models. This will provide a more solid theoretical foundation and experimental basis for future clinical applications.

In conclusion, our study identifies USP48 as a critical substrate for caspase-3 during drug-induced apoptosis and differentiation in AML cells. The cleavage of USP48 by caspase-3 leads to the degradation of its N-terminal fragment, sensitizing AML cells to apoptotic signals and enhancing the efficacy of chemotherapy. These findings underscore the potential of USP48 as a therapeutic target in AML and pave the way for future studies to explore the clinical implications of USP48 inhibition in cancer treatment.

## Data Availability

The datasets used and/or analyzed are available from the corresponding author on reasonable request.

## Abbreviations

AML: Acute myeloid leukemia
ATRA: all-trans-Retinoic acid
DUBs: Deubiquitinases
USPs: ubiquitin-specific proteases
HHT: Homoharringtonine
USP48: ubiquitin-specific peptidase 48
